# Supplementary material for: Generalization of long-range machine learning potentials in complex chemical spaces
Source: Digit Discov. 2026 Apr 13;5(5):2195–208. doi: 10.1039/d5dd00570a (PMC13093489; doi:10.1039/d5dd00570a)
Supplement: DD-005-D5DD00570A-s001 [file DD-005-D5DD00570A-s001.pdf]

Supplementary Information (SI) for Digital Discovery.
This journal is © The Royal Society of Chemistry 2026

Supplementary Information: Generalization of Long-Range Machine Learning Potentials in Complex Chemical Spaces

Michał Sanocki,*[a] and Julija Zavadlav[a]

[a] *Multiscale Modeling of Fluid Materials, Department of Engineering Physics and Computation, TUM School of Engineering and Design, Technical University of Munich, Germany; E-mail: julija.zavadlav@tum.de*

1 Preparation of subsplits of ODAC25 and OMOL25 datasets

For the OMOL25 dataset, we focused on the metal–organic subsplit as those molecules should be related to MOFs; however, this dataset does not have associated SMILES or other molecular identifiers, which disables simple grouping by source molecule [1]. To address this, we identified molecules based on the root directory contained in the dataset metadata. For each such root, we compared all associated structures and retained only the lowest-energy configuration, thereby constructing a representative set of candidate ground-state structures. This procedure was necessary to reduce redundancy and to enable consistent analysis across the dataset; however, it does not guarantee that the selected structures correspond to the true ground state of the molecules. For ODAC25, the process was simpler than for OMOL25 since each structure comes with an MOF ID. Structures from the re-relaxations of the empty MOF subset were grouped by this ID, and the lowest-energy configuration was retained as the representative. The resulting subset of the OMOL25 consists of 38263 unique molecules (max 350 atoms), and ODAC25 of 2089 MOFs (max 616 atoms). As a result, we decided to include the 10 lowest energy conformations for each MOF in the ODAC25 subset, which also allowed us to evaluate our method on a test case with multiple points per molecule.

2 Biased splits

It is worth noting that the UMAP-reduced spaces differ significantly across the three datasets. The ODAC25 dataset consists of many small, isolated clusters; OMOL25 has two large components with numerous additional isolated points; and QMOF contains many points in closer proximity to each other, though not as dense as in OMOL. These differences can result from several factors, such as the size of the dataset, intrinsic structural properties, characteristics of the SOAP descriptor, limitations of UMAP dimensionality reduction, and the presence of rare structures. The results also suggest that the ODAC25 dataset spans the most diverse chemical space. In QMOF, size appears to be relatively evenly distributed across the descriptor space, whereas in ODAC25 and especially OMOL, there is a significant concentration of larger molecules in certain areas of chemical space.

As seen in the figures above, in some max separation splits, the resulting datasets are very similar, as the final split depends mostly on the choice of starting point (the rest of the algorithm is deterministic), and it is possible that similar starting points will result in very similar final splits.

Table 1 SOAP descriptor parameters used in this work, same as in reference[2]

**Parameter**	**Value**
Cutoff radius r_{cut} (Å)	4.0
Gaussian width σ (Å)	0.10
Radial basis size n_{max}	9
Angular basis size l_{max}	9
Radial basis type	GTO

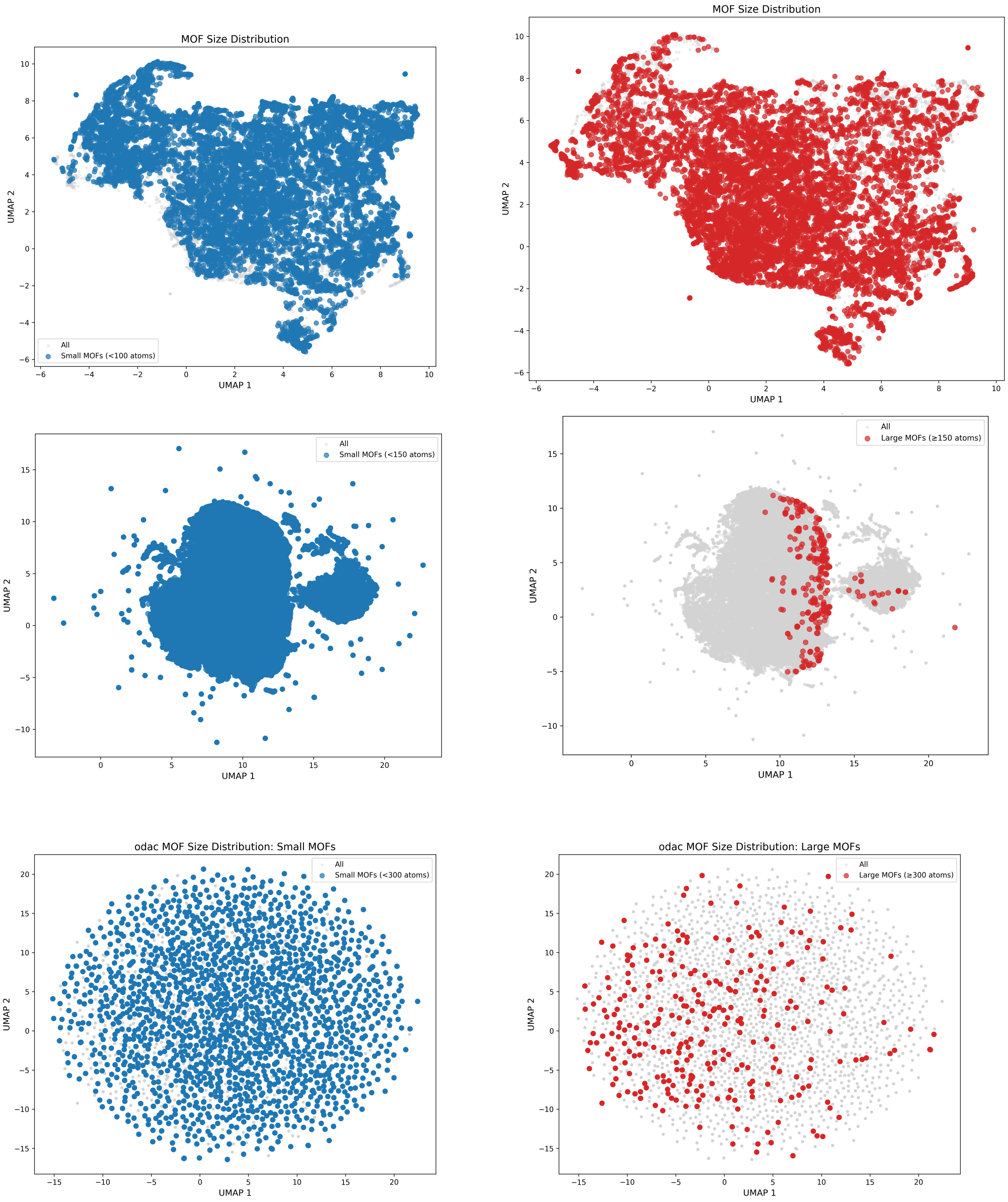

Fig. 1 UMAP-based visualisations of Small-Large molecule split for all datasets. Left - Small set (blue), Right - Large set (red), grey points represent the full dataset. Molecules with more than 150 atoms in OMOL, 100 in QMOF, and 300 in ODAC25 were labelled as large. Splitting into small and large molecules was adjusted so that a comparable number of molecules would be present in the test set in all splits.

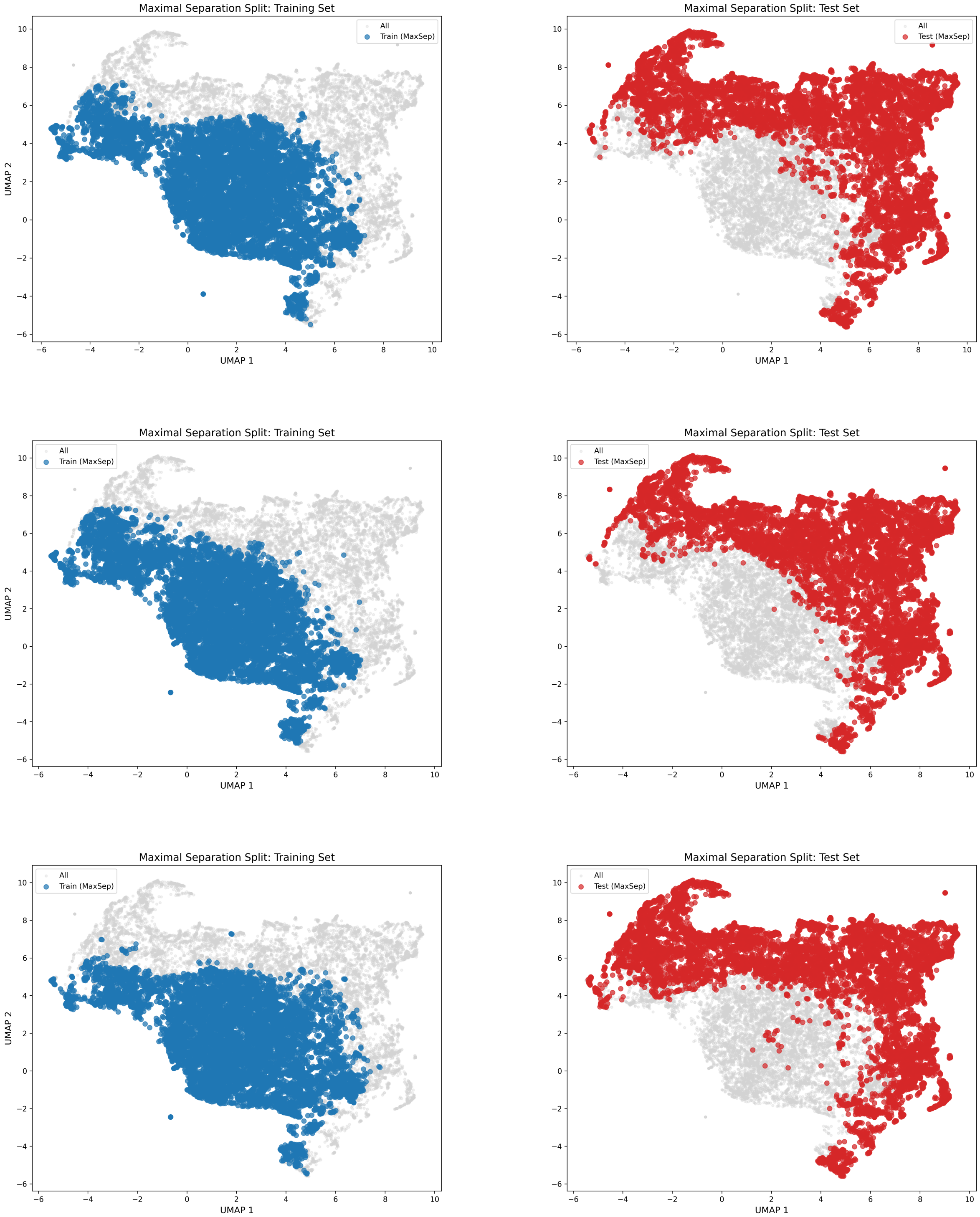

Fig. 2 UMAP-based visualisations of three maximum separation splits for QMOF dataset. Left - training set (blue), right - test set (red), grey points correspond to the full dataset.

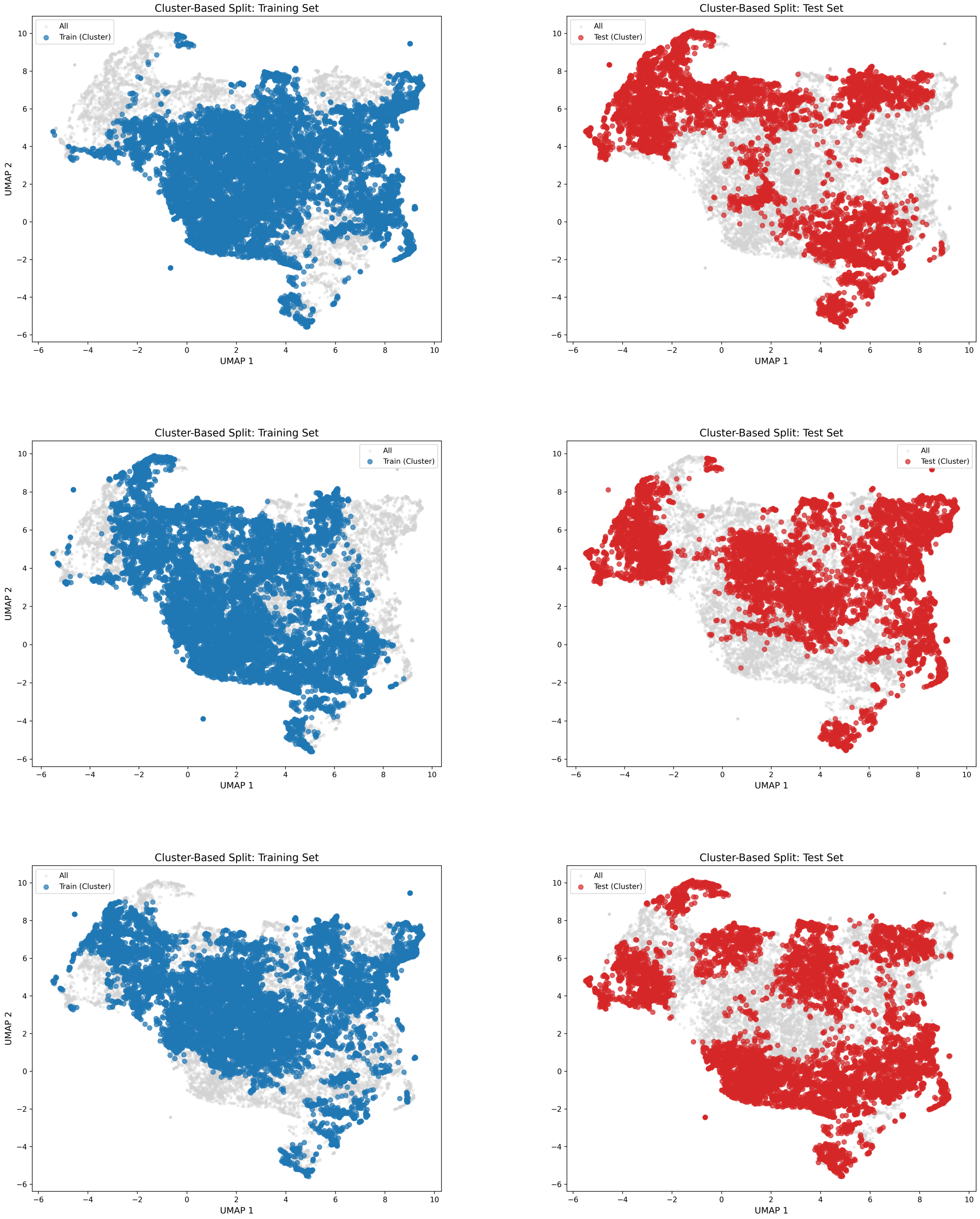

Fig. 3 UMAP-based visualisations of three cluster separation splits for QMOF dataset. Left - training set (blue), right - test set (red), grey points correspond to the full dataset.

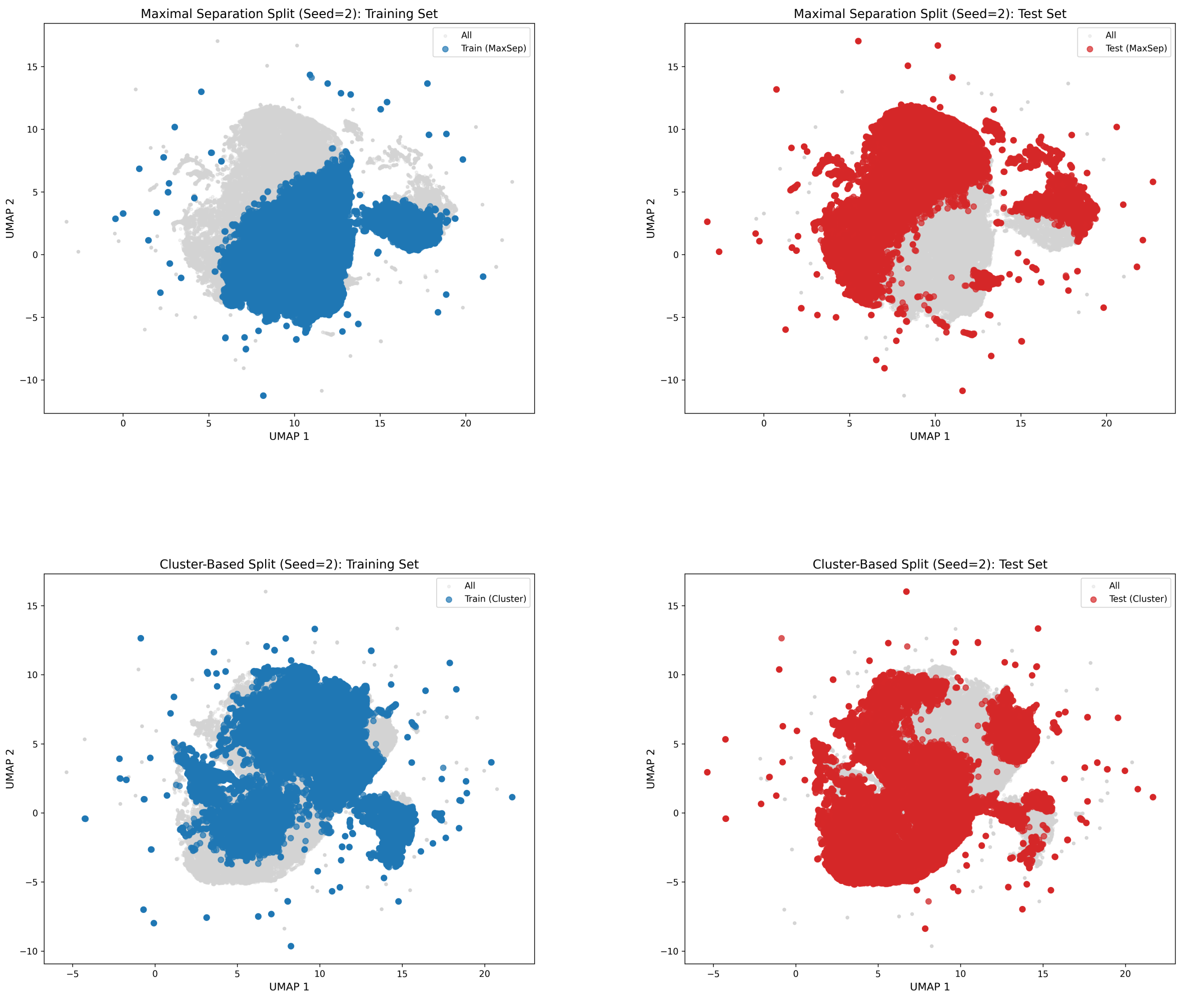

Fig. 4 UMAP-based visualisations of cluster and maximum separation split for OMOL25 dataset. Left - training set (blue), right - test set (red), grey points correspond to the full dataset.

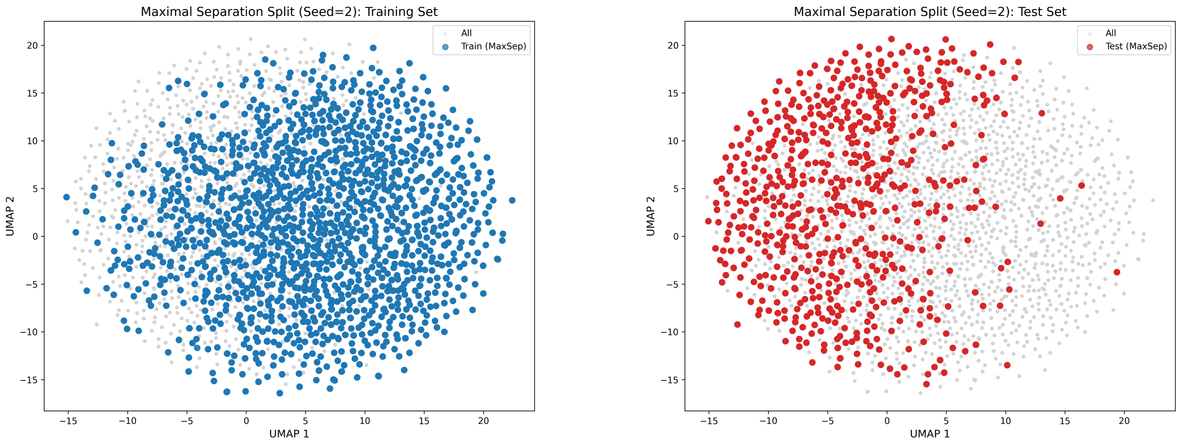

Fig. 5 UMAP-based visualisations of maximum separation split for ODAC25 dataset. Left - training set (blue), right - test set (red), grey points correspond to the full dataset.

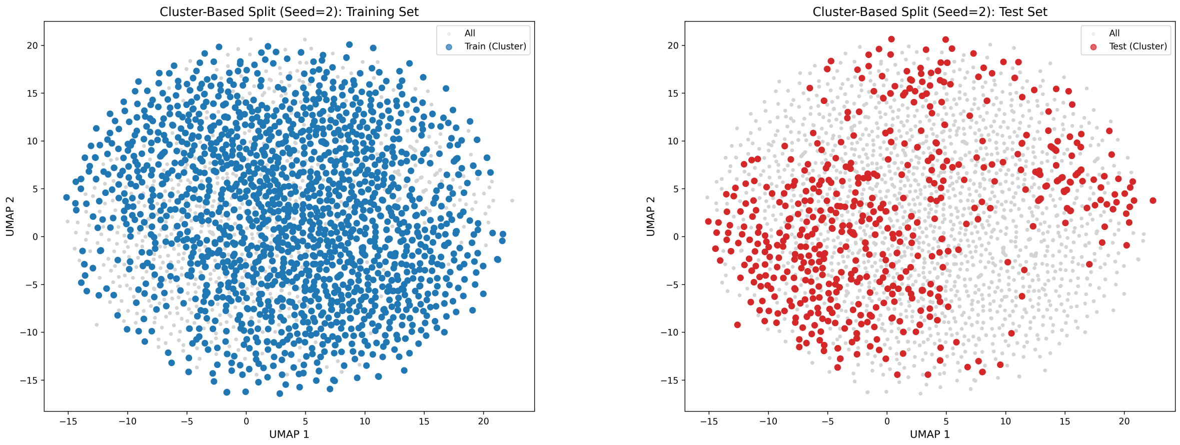

Fig. 6 UMAP-based visualisations of cluster split for ODAC25 dataset repetitions. Left - training set (blue), right - test set (red), grey points correspond to the full dataset.

3 Data Preprocessing

In the ODAC25 and QMOF datasets, periodic images were corrected according to the *Minimum Image Convention* (MIC) to ensure all pairwise interactions within the cutoff radius are properly captured. Structures were replicated along box dimensions if the cutoff exceeded half the shortest box vector, guaranteeing no interactions were truncated.

Since reference energies include large negative offsets, we applied species-dependent constant shifts U_s to obtain corrected target energies:

$$\hat{U}_i = U_i - \sum_{s=1}^{S} U_s N_{s,i} \tag{1}$$

where $N_{s,i}$ is the number of atoms of species s in sample i. The shifts U_s were determined by ridge regression on each dataset.

4 Training

Models were trained using the Force Matching method[3,4], minimizing the loss

$$\mathscr{L}(\theta) = \frac{1}{D}\sum_{i=1}^{D}\Big[\gamma_U \|U_\theta(\boldsymbol{R}_i) - \hat{U}_i\|^2 + \tfrac{\gamma_F}{3N_i}\|\boldsymbol{F}_\theta(\boldsymbol{R}_i) - \hat{\boldsymbol{F}}\|^2 + \tfrac{\gamma_Q}{N_i}\|\boldsymbol{Q}_\theta(\boldsymbol{R}_i) - \hat{\boldsymbol{Q}}\|^2\Big], \tag{2}$$

where $U, \boldsymbol{F}, \boldsymbol{Q}$ are model predictions and $\hat{U}, \hat{\boldsymbol{F}}, \hat{\boldsymbol{Q}}$ are references. We used ADAM[5] with a polynomial learning-rate schedule and weight decay. Target weights $\gamma_U, \gamma_F, \gamma_Q$ were problem-specific. Model selection was based on the lowest validation error.

5 Hyperparameters

Table 2 Model hyperparameters for QMOF, OMOL, and ODAC25 datasets. Coulomb cutoff *max* refers to a cutoff greater than the largest distance between any pair of atoms in the dataset. The number of Interaction Layers (Allegro), Message Passing (MACE), or Interaction Blocks (DimeNet++) is reported. Dashed lines indicate that no combination of dataset and model architecture was trained.

	QMOF	OMOL	ODAC
**Allegro**[6]			
r_{cut} GNN (nm)	0.45	0.50	0.45
r_{cut} [a,b] LR (nm)	0.50	2.50	0.50
Irreps	$64 \times 1e + 64 \times 1o$	$64 \times 1e + 64 \times 1o$	$64 \times 1e + 64 \times 1o$
MLP dim	64	128	64
MLP layers	2	3	2
Embedding dim	128	128	128
Interaction layers	(2, 1)	(2, 1)	(2, 1)
γ_U	0.0001	0.005	1
γ_F	–	0.1	100
γ_Q [c]	5	10.0	–
Epochs	80	100	100
Batch size	6	12	4
**MACE**[7]			
r_{cut} GNN (nm)	0.45	0.50	0.45
r_{cut} Coul. (nm)	0.50	2.50	0.50
Irreps	$64 \times 0e + 128 \times 1o + 64 \times 1e$	$64 \times 0e + 64 \times 1o$	$64 \times 0e + 64 \times 1o$
$\text{MLP}_{\mathscr{R}}$ dim	128	128	128
$\text{MLP}_{\mathscr{R}}$ layers	3	3	3
MP layers	2	2	2
γ_U	0.01	0.002	1
γ_F	–	0.5	100
γ_Q [c]	20.0	1000.0	100.0
Epochs	70	100	100
Batch size	6	12	4
**DimeNet++**[8]			
r_{cut} GNN (nm)	0.45	–	0.45
Embedding dim	128	–	128
Interaction blocks	2	–	2
# RBF	6	–	6
# SBF	7	–	7
γ_U	1	–	0.05
γ_F	–	–	100
Epochs	140	–	100
Batch size	12	–	4

[a] CELLI = Coulomb cutoff, combined with long-range electrostatics via SPME method.[9]
[b] EFA – `era_max_length`, which ensures rotational invariance.
[c] For models with CELLI, otherwise 0.

Table 3 Number of parameters (in millions) for different models across ODAC, QMOF, and OMOL datasets. Allegro architectures are identical for ODAC and QMOF.

Model	ODAC	QMOF	OMOL
Allegro	0.16	0.16	–
Allegro CELLI	0.22	0.22	0.20
Allegro EFA	0.16	0.16	–
Allegro C EMB	–	–	0.24
MACE	2.49	2.60	–
MACE MP4	4.70	8.35	–
MACE CELLI	2.57	2.69	2.57
MACE EFA	2.56	2.69	–
MACE C EMB	–	–	2.50

Table 4 Hyperparameters for the MACE-LES models. Due to the increased computational cost associated with using the PyTorch implementation compared to JAX, MACE LES models have different hyperparameters.

**Parameter**	**MACE-LES S**	**MACE-LES L**	**MACE-LES QMOF**
E_0 offset	average	average	average
Number of channels	64	64	64
Hidden irreps	$64 \times 0e + 64 \times 1o$	$64 \times 0e + 64 \times 1o$	$64 \times 0e + 64 \times 1o$
$r_{\max}$ (Å)	5	4.5	4.5
Max spherical order $L_{\max}$	1	2	2
Number of interactions (MP layers)	2	2	2
Correlation order	3	2	2
Batch size	10	2	4
Max epochs	150	150	100
Energy weight γ_U	10	10	10
Force weight γ_F	10000	10000	0
EMA	enabled	enabled	enabled
EMA decay	0.99	0.99	0.99
Scheduler patience	15	15	15
Early-stopping patience	50	50	50

Table 5 Default EFA parameters used in this work. ERA - Euclidean Rope Attention. For details see[10]

**Parameter**	**Value**
EFA iterations used	[0, 1]
Number of Lebedev grid points	50
Number of features for query/key vectors	16
Number of features for value vectors	32
Activation function	`silu`
ERA Number of frequencies	8
ERA Maximum frequency	π
ERA Maximum tensor degree	0

6 Model Performance Table

Table 6 QMOF test-set RMSE for energy (RMSE U) with standard deviation (Std).

Model family	Variant	Split	RMSE U [meV/atom]	
Allegro	Baseline	Small/Large	18.79	0.58
		Cluster	28.71	5.72
		Rand	23.44	0.58
		Maxsep	64.36	4.08
	CELLI	Small/Large	14.15	1.61
		Cluster	17.46	1.58
		Rand	14.91	0.68
		Maxsep	26.71	1.77
	EFA	Small/Large	11.44	1.55
		Cluster	20.54	1.43
		Rand	18.93	0.98
		Maxsep	58.70	2.31
DimeNet++	Baseline	Small/Large	11.85	1.93
		Cluster	44.00	21.64
		Rand	17.81	4.54
		Maxsep	25.22	1.87
MACE	Baseline	Small/Large	11.68	1.97
		Cluster	16.84	1.11
		Rand	10.75	0.77
		Maxsep	41.61	2.09
	CELLI	Small/Large	13.61	1.79
		Cluster	17.47	1.20
		Rand	11.26	1.03
		Maxsep	23.84	1.17
	EFA	Small/Large	13.08	2.19
		Cluster	16.60	1.03
		Rand	12.60	1.20
		Maxsep	24.56	0.93
	MP4	Small/Large	14.31	0.53
		Cluster	38.33	1.45
		Rand	12.17	0.39
		Maxsep	151.03	3.23

Table 7 ODAC25 model performance by model family and data splits.

Model family	Variant	Split	MAE U [meV/atom]	MAE F [meV/Å
DimeNet++	Baseline	Small/Large	40.60	171.15
		Cluster	49.89	26.18
		Rand	38.93	27.95
		Maxsep	47.74	35.18
MACE	EFA	Small/Large	363.44	40.82
		Cluster	30.41	32.78
		Rand	63.42	29.99
		Maxsep	27.38	33.44
	MP4	Small/Large	63.78	52.31
		Cluster	29.34	24.99
		Rand	33.70	33.89
		Maxsep	37.36	33.45
	CELLI	Small/Large	94.32	65.82
		Cluster	31.70	26.16
		Rand	33.88	39.95
		Maxsep	29.52	29.02
	Baseline	Small/Large	71.39	42.74
		Cluster	26.06	27.31
		Rand	32.10	32.04
		Maxsep	25.70	30.40
Allegro	CELLI	Small/Large	45.26	42.32
		Cluster	80.37	27.76
		Rand	79.24	30.47
		Maxsep	74.07	33.27
	EFA	Small/Large	45.22	92.15
		Cluster	50.86	22.71
		Rand	47.02	29.12
		Maxsep	52.99	32.91
	Baseline	Small/Large	52.02	21.58
		Cluster	79.60	22.12
		Rand	73.83	30.57
		Maxsep	74.34	33.17

Table 8 OMO25 model performance grouped by models and data splits. C EMBEDDING denotes charge-embedding variants.

Model family	Variant	Split	MAE U [meV/atom]	MAE F [meV/Å
MACE	CELLI	Small/Large	21.58	75.21
		Cluster	70.37	77.09
		Rand	57.00	72.00
		Maxsep	66.60	76.59
	C Embedding	Small/Large	28.70	73.39
		Cluster	49.38	78.25
		Rand	42.00	74.19
		Maxsep	48.70	75.80
Allegro	CELLI	Small/Large	20.43	96.57
		Cluster	26.51	88.81
		Rand	26.03	86.29
		Maxsep	33.18	89.22
	C Embedding	Small/Large	9.80	86.84
		Cluster	25.06	87.13
		Rand	19.63	81.95
		Maxsep	23.99	88.34

7 Error on SOAP descriptor space

Interestingly, there are areas of chemical space that exhibit similar trends across all models, showing that many architectures show similar bias. This is less pronounced for models with EFA and CELLI schemes, which is in agreement with our expectations.

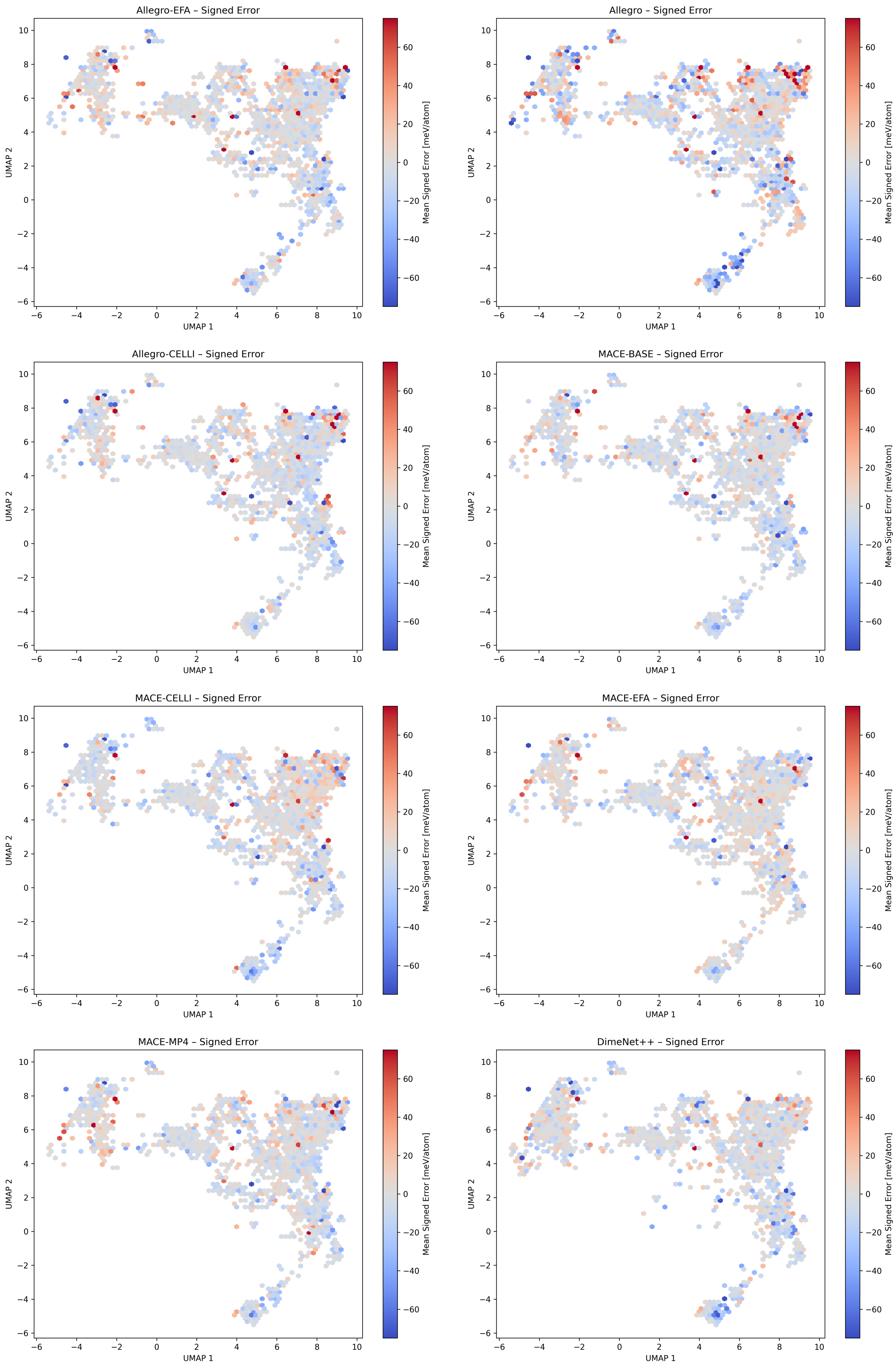

Fig. 7 Error on the chemical space defined by UMAP-reduced SOAP descriptors of the QMOF dataset for models used in this study on the max separation split.

8 Replication of LES results

To ensure that we can reproduce the results presented by King *et al.*, we tried to recreate results for the dipeptides benchmark based on the provided data. The results for the CACE model are indistinguishable from those presented by King *et al.*; to our knowledge, results for MACE were not made available. This suggests that issues with poor performance of LES models on charge inference in difficult datasets likely result from shortcomings of the LES framework. Due to the high computational cost of applying CACE=LES to QMOF and ODAC datasets (model size grows with the number of species and body order), only the MACE implementation was utilized in the benchmarks.

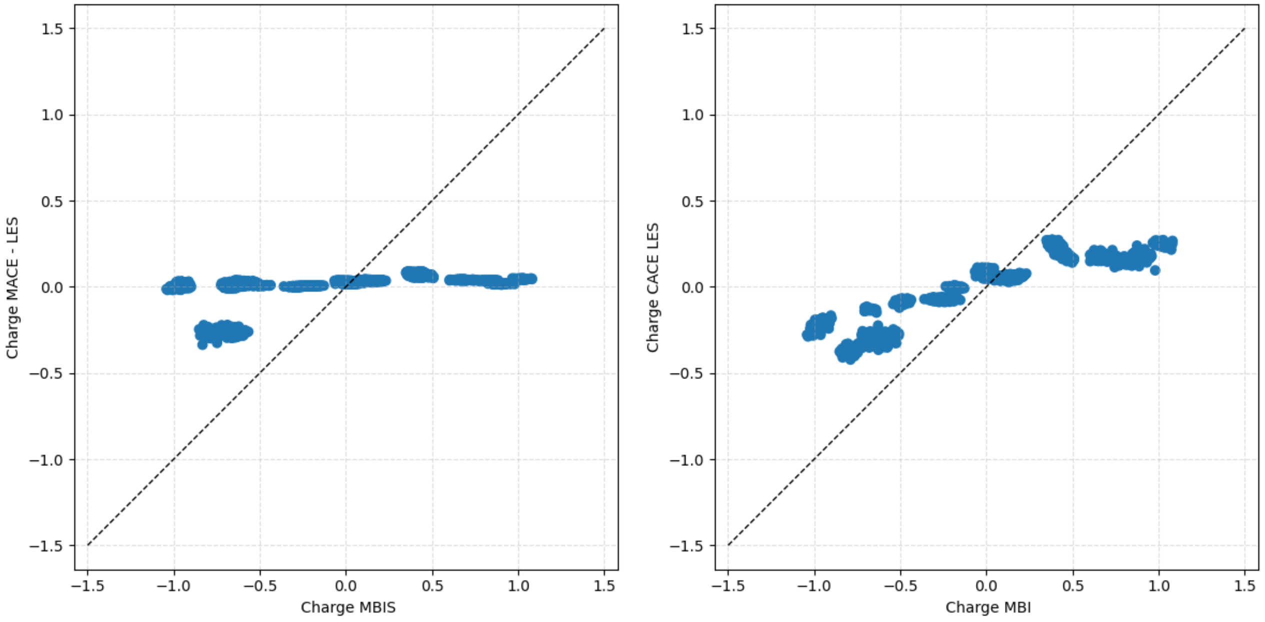

Fig. 8 Charge parity plots for dipeptide benchmark for King *et al.*

9 BEC Computation

We compute the Born effective charge (BEC) tensor for each atom as the derivative of the macroscopic polarization $\mathbf{P}$ with respect to atomic displacement:

$$Z^*_{i,\alpha\beta} = \frac{\partial P_\alpha}{\partial r_{i\beta}}, \tag{3}$$

where $\alpha, \beta \in \{x, y, z\}$ and $r_{i\beta}$ denotes the β-component of the position of atom i.

For non-periodic systems, polarization is evaluated from predicted atomic charges q_i and positions $\mathbf{r}_i$,

$$\mathbf{P} = \sum_{i=1}^{N} q_i \, \mathbf{r}_i. \tag{4}$$

In periodic systems, direct summation is ill-defined; we use a Berry-phase-inspired formulation based on the phase of fractional coordinates $\mathbf{r}_i^{\text{frac}} = \mathbf{r}_i \mathbf{h}^{-1}$,

$$\mathbf{P} = \frac{\mathbf{h}}{i2\pi} \left(\sum_{i=1}^{N} q_i \, e^{i2\pi \mathbf{r}_i^{\text{frac}}} \right), \tag{5}$$

followed by dephasing, after which the real component of $\mathbf{P}$ is used.

The BEC tensor is obtained via automatic differentiation of the real polarization:

$$Z^*_{i,\alpha\beta} = \frac{\partial \, \mathrm{Re}[P_\alpha]}{\partial r_{i\beta}}. \tag{6}$$

Datasets padded to a maximum atom count are handled by masking; padded atoms have their charges and positions zeroed and produce zero-valued BEC tensors. The computation is vectorized with `vmap` and accelerated with `jit` in JAX.

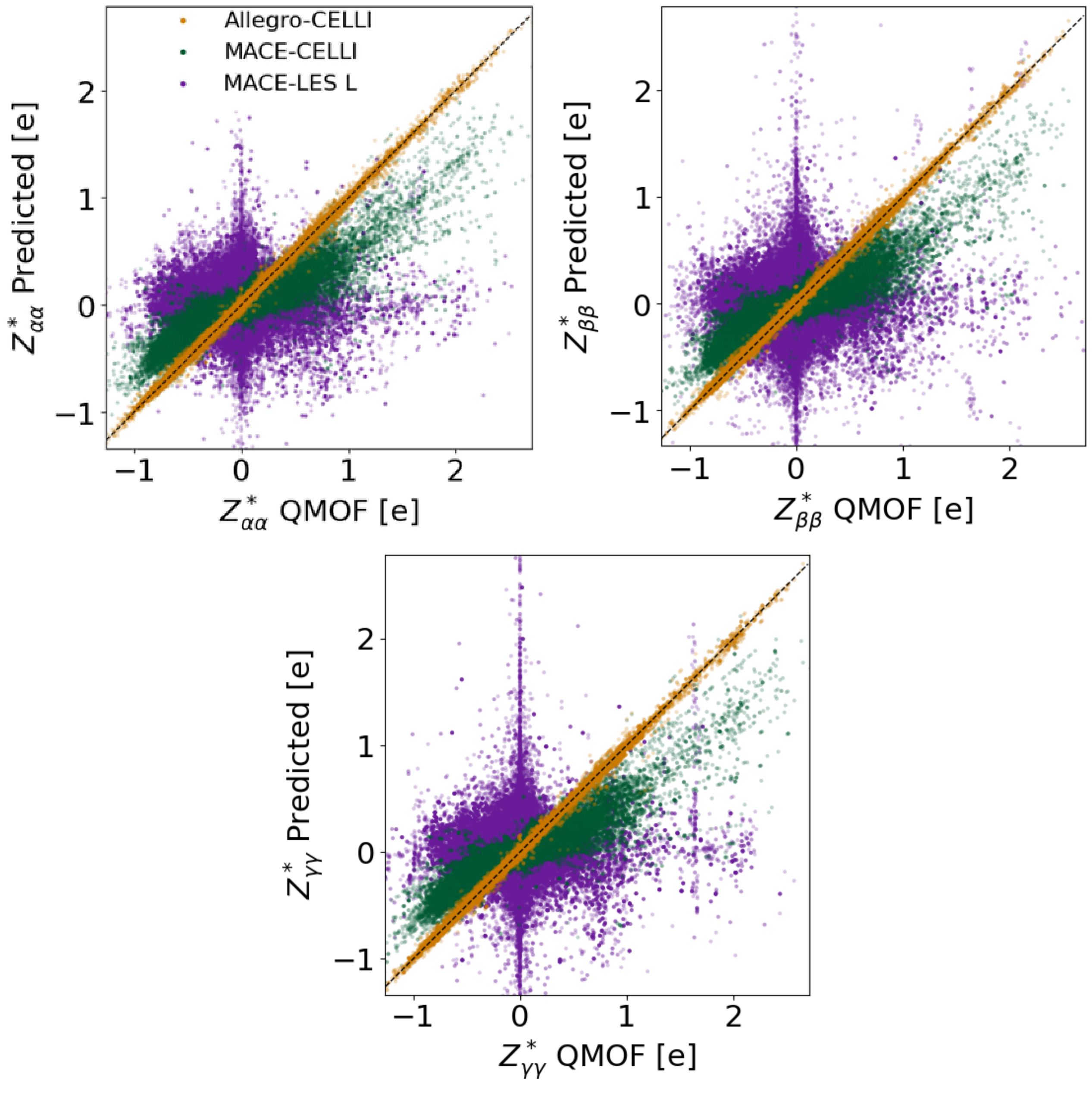

Fig. 9 Born Effective Charge Prediction. Parity plot of diagonal elements of predicted Born effective charge tensors for MACE-LES models trained on a random split of the QMOF dataset.

References

1 D. S. Levine, M. Shuaibi, E. W. C. Spotte-Smith, M. G. Taylor, M. R. Hasyim, K. Michel, I. Batatia, G. Csányi, M. Dzamba, P. Eastman, N. C. Frey, X. Fu, V. Gharakhanyan, A. S. Krishnapriyan, J. A. Rackers, S. Raja, A. Rizvi, A. S. Rosen, Z. Ulissi, S. Vargas, C. L. Zitnick, S. M. Blau and B. M. Wood, *The Open Molecules 2025 (OMol25) Dataset, Evaluations, and Models*, 2025, `https://arxiv.org/abs/2505.08762`.

2 A. S. Rosen, S. M. Iyer, D. Ray, Z. Yao, A. Aspuru-Guzik, L. Gagliardi, J. M. Notestein and R. Q. Snurr, *Matter*, 2021, **4**, 1578–1597.

3 F. Ercolessi and J. B. Adams, *Europhysics Letters (EPL)*, 1994, **26**, 583–588.

4 P. Fuchs, S. Thaler, S. Röcken and J. Zavadlav, *Computer Physics Communications*, 2025, **310**, 109512.

5 D. P. Kingma and J. Ba, *Adam: A Method for Stochastic Optimization*, 2017.

6 A. Musaelian, S. Batzner, A. Johansson, L. Sun, C. J. Owen, M. Kornbluth and B. Kozinsky, *Nature Communications*, 2023, **14**, 579.

7 I. Batatia, D. P. Kovács, G. N. C. Simm, C. Ortner and G. Csányi, *MACE: Higher Order Equivariant Message Passing Neural Networks for Fast and Accurate Force Fields*, 2023, `https://arxiv.org/abs/2206.07697`.

8 J. Gasteiger, S. Giri, J. T. Margraf and S. Günnemann, *Fast and Uncertainty-Aware Directional Message Passing for Non-Equilibrium Molecules*, 2022.

9 P. Fuchs, M. Sanocki and J. Zavadlav, *Learning non-local molecular interactions via equivariant local representations and charge equilibration*, 2025.

10 J. T. Frank, S. Chmiela, K.-R. Müller and O. T. Unke, *Euclidean Fast Attention: Machine Learning Global Atomic Representations at Linear Cost*, 2024.

11 D. S. King, D. Kim, P. Zhong and B. Cheng, *Nature Communications*, 2025, **16**, 8763.
